# The Performance of *Malaysian Journal of Medical Sciences* in 2020 and the Impact of COVID-19 on the Trend of Manuscript Submissions

**DOI:** 10.21315/mjms2021.28.4.1

**Published:** 2021-08-26

**Authors:** Zulkapli Nour Azimah, Jafri Malin Abdullah

**Affiliations:** 1 Malaysian Journal of Medical Sciences, Universiti Sains Malaysia, Pulau Pinang, Malaysia; 2 Malaysian Journal of Medical Sciences, Universiti Sains Malaysia, Kubang Kerian, Kelantan, Malaysia

**Keywords:** journal performance, submission trend, COVID-19, recognition

## Abstract

In this Editorial, we report on the trend of manuscript submissions during the peak of COVID-19 pandemic in 2020. The total monthly new manuscript submissions from 2018 to 2020 were compared to examine the overall trends in submission and publication activities.

Apart from that, we also report on the changes in the journal’s administration and the external recognition received by the journal.

## Introduction

The emergence of COVID-19 as a global pandemic in early 2020 and the impose of movement control order by the government has affected many aspects of both personal and professional lives of people around the world.

## Performance

### New Manuscript Submissions Trend

**Figure 1:**
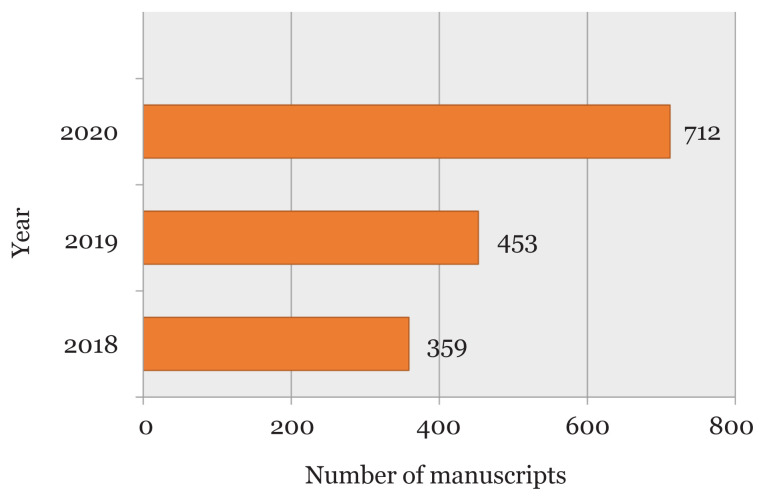
The total of new MJMS manuscript submissions from 2018–2020 Source: https://mc.manuscriptcentral.com/maljms

In year 2020, a total of 712 new manuscripts were submitted to the *Malaysian Journal of Medical Sciences* (MJMS)’ electronic manuscript submission system (Figure 1). In average, this is a significant increase compared to the preceding years which is presumably due to COVID-19 pandemic when many researchers may be spending more time writing and preparing manuscripts than before the pandemic began. As suggested by Alkhouri et al. (1), COVID-19 may have altered the work focus of some researchers towards manuscript production and publication. This increase also is due to the submission of manuscript related to COVID-19.

With people having to work at home, it took a while to adopt to this ‘new normal.’ Due to the movement control order imposed by the government of Malaysia and many other countries, the research community have to work from home when most of research laboratories were shut down starting in March 2020.

**Figure 2:**
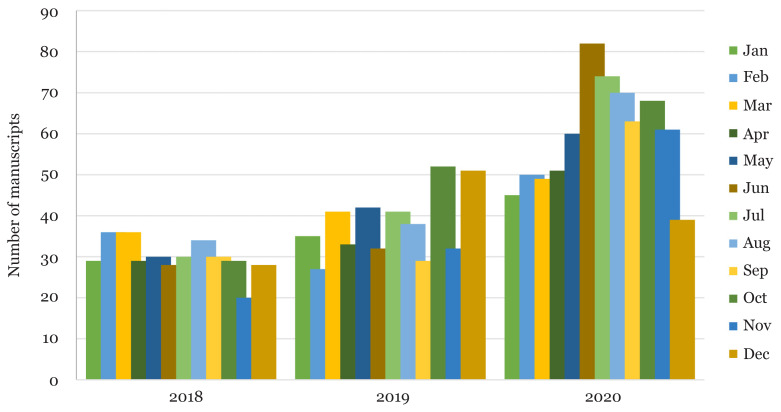
New manuscript submissions trend compared by year and month Source: https://mc.manuscriptcentral.com/maljms

As shown in Figure 2, number of submissions increased almost imperceptible in March 2020 but in ensuing months, especially in June 2020, MJMS received 82 new manuscripts. The MJMS journal administrator was being challenged with this very significant number of new manuscript submissions. Besides hustling with other tasks related to publication, this person is responsible for processing all new submissions, ensuring that the manuscripts are prepared according to the journal guidelines and attending authors correspondence. So, needless to say, the feeling of heavy weight of these situation was at most.

**Figure 3:**
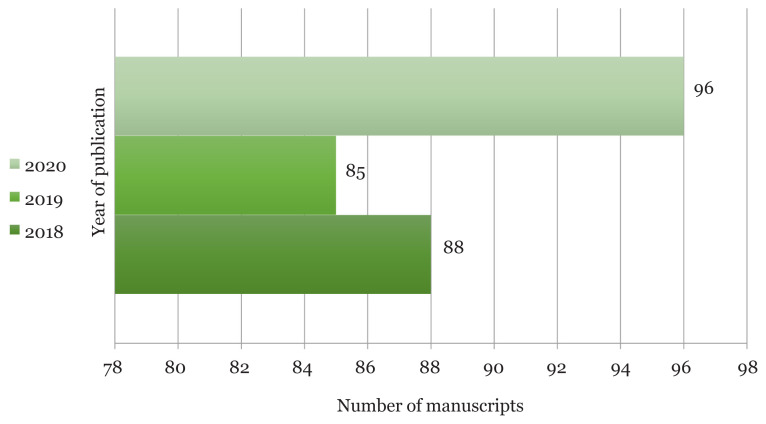
Manuscripts publication compared by year Source: https://mc.manuscriptcentral.com/maljms

Despite the hard situation during the peak of pandemic, the workflow of publication process was on schedule. As shown in Figure 3, MJMS managed to publish 96 accepted manuscripts in 2020, apart from the publication of abstracts as Special Issues (acquired from a conference) and abstract of theses approved for PhD, MMed and MSc.

Due to the number of manuscripts received was likely out of balance with the number of the editorial board members, especially statistical editors, three names have been nominated and accepted as new members of MJMS. Dr Anis Kausar Ghazali, Dr Nur Syahmina Rasudin and Dr Wan Arfah Nadiah Wan Abdul Jalil were the new addition to the statistical editor team effective 1 January 2020.

### Altmetric Scores and External Recognition

The MJMS’s Impact Score for 2020 was 1.39, increased by a factor of 0.48 and approximate percentage change is 52.75% when compared to preceding year 2019, which shows a rising trend. Other scores which might of interest are:

SCImago Journal Rank (SJR): 0.394
CiteScore: 2.0
H-index: 25

The Professional Evaluation Panel of the Majlis Penerbitan Ilmiah Malaysia (MAPIM)-Kementerian Pengajian Tinggi (KPT) Award 2019 has chosen a manuscript published in the first volume of MJMS 2019 to win the Best Journal Manuscript Award (Science, Technology and Medical) International Index. This event is held annually but due to COVID-19 pandemic, it has been postponed to 30 March 2021 and was conducted via Cisco Webex and Facebook Live MAPIM. The details of the winning manuscript were as follows:

**Table:** 

Manuscript title:	Ameliorative effects of *Aquilaria malaccencis* leaves aqueous extract on reproductive toxicity induced by cyclophosphamide in male rats
Authors:	Redzuan Nul Hakim Abdul Razak, Faridah Ismail, Muhammad Lokman Md Isa, Azantee Yazmie Abdul Wahab, Hussin Muhammad, Roszaman Ramli *and Raja Arif Shah Raja Ismail*
*Journal:*	*The Malaysian Journal of Medical Sciences*, Volume 26, No. 1, 2019
Publisher:	Universiti Sains Malaysia Press
